# Editorial: Multiple organ dysfunctions in perioperative critical illness and the prognosis after anesthesia

**DOI:** 10.3389/fmed.2023.1305053

**Published:** 2023-10-23

**Authors:** Ruixue Liu, Qiao Guo, Jihad Mallat, Chenyang Duan

**Affiliations:** ^1^Department of Anesthesiology, The Second Affiliated Hospital of Chongqing Medical University, Chongqing, China; ^2^Department of Critical Care Medicine, Critical Care Institute, Cleveland Clinic Abu Dhabi, Abu Dhabi, United Arab Emirates

**Keywords:** critical care, anesthesia, multiple organ dysfunctions, MODS, inflammation

During the perioperative period, patients may encounter a range of stress factors, such as blood loss, hypoperfusion, inflammatory responses, and multi-organ dysfunctions (MODS). The combined effects of these elements can profoundly shape a patient's post-anesthesia prognosis. With this in mind, we are excited to introduce a special edition Research Topic: “*Multiple Organ Dysfunctions in Perioperative Critical Illness and the Prognosis After Anesthesia*”. This theme endeavors to gather insights on the incidence and underlying mechanisms of MODS, explore novel monitoring/evaluation techniques and early biomarkers for organ dysfunction during the perioperative phase, and delve into the impact of anesthetic drugs on organ functions during this crucial time.

Regarding the mechanisms of multi-organ dysfunction in critical disease, Zhao et al. examined the influence of the HIF-1α/BNIP3L signaling pathway on sepsis-associated encephalopathy (SAE). Their findings indicated that the activation of the HIF-1α/BNIP3L pathway is associated with cognitive impairment. Concurrently, Wang Y. et al. investigated the role of Cathepsin B (CTSB) in the context of sepsis-induced acute kidney injury. They ascertained that mitochondrial apoptosis plays a pivotal role in the progression of sepsis-related kidney damage, yet CTSB has the potential to counteract this adverse effect. Building on this insight, they suggested that CTSB inhibition could emerge as a promising therapeutic avenue for mitigating sepsis-induced acute kidney injury during the perioperative period.

In this topic, a multitude of efficacious monitoring and evaluation measures for organ dysfunction have been unveiled. Xiao et al. honed in on the impact of individualized positive end-expiratory pressure (PEEP) under the guidance of electrical impedance tomography on the distribution of pulmonary ventilation in patients undergoing abdominal thermal perfusion chemotherapy. Their findings illuminate a novel individualized therapeutic approach to enhance lung functionality in perioperative patients. Meanwhile, Li et al. explored the optimal oxygen therapy strategy for perioperative patients diagnosed with SAE. Their research elucidated a significant correlation between oxygen therapy modalities and the incidence of SAE, leading to the formulation of a targeted oxygen therapy objective for SAE. Lastly, through a comprehensive network meta-analysis, Wang J. et al. compared various mechanical ventilation strategies in obese patients. They determined that volume-controlled ventilation, when combined with individualized PEEP and a recruitment maneuver, stood out as the most effective method to bolster the PaO_2_/FiO_2_ ratio, thus offering a tangible guideline and treatment approach to expedite postoperative recovery in obese individuals.

In recent years, there has been a marked surge in research focusing on early biomarkers for organ dysfunction in critical illnesses. Notably, efforts geared toward evaluating and pinpointing effective biomarkers, especially through the lenses of organelle damage and mitochondrial metabolic anomalies, have gained considerable traction. In this context, the studies of Hao et al. and Shu Q. et al. delved into the potential of mitochondrial-associated genes in the early diagnosis of sepsis. Their findings underscored the significant diagnostic merit of assessing mitochondrial function when gauging organ damage and determining risk stratification in sepsis scenarios. Concurrently, Zhou et al. embarked on a quest to ascertain the role of genes linked to the endoplasmic reticulum in the precursory diagnosis of sepsis. Their insights pave the way for prospective therapeutic and diagnostic strategies for multi-organ dysfunction syndrome (MODS) arising from sepsis. Additionally, She et al. explored the influence of genes associated with lipid metabolism on immune modulation in sepsis. Their contributions shed light on potential therapeutic avenues that, by adjusting metabolism, can bolster immune functionality in septic patients.

Furthermore, several studies have scrutinized and analyzed the predictive accuracy of extant clinical indicators concerning perioperative organ function and patient outcomes during the perioperative phase. Specifically, Tian et al. assessed the repercussions of preoperative inflammation on primary outcomes post-cardiac valve surgery. They discerned that preoperative inflammatory markers, including levels of C-reactive protein, erythrocyte sedimentation rate, and the neutrophil-to-lymphocyte ratio, hold predictive value for clinical outcomes following heart valve procedures. Zhu, Bi, Yu et al. gauged the prognostic significance of preoperative high-sensitivity cardiac troponin T (hs-cTnT). Their findings revealed a notable association between elevated preoperative hs-cTnT concentrations and increased long-term mortality following non-cardiac surgeries. This underscores the potential of preoperative hs-cTnT as a pivotal biomarker for evaluating the long-term prognosis of patients post non-cardiac surgeries, offering foundational insights for preoperative risk stratification and ensuing patient care and management. In a separate study, Zhu, Bi, Liu et al. evaluated the prognostic efficacy of the pre-surgical neutrophil-to-lymphocyte ratio (NLR) on postoperative mortality and complications. A notable revelation from their study is the establishment of a threshold NLR value of 3.6, which is markedly associated with escalated in-hospital mortality rates and increased ICU admissions post-surgery. Shu B. et al. delved into the predictive potential of perioperative NLR shifts as biomarkers for chronic post-surgical pain (CPSP) and quality of life. Analyzing data from 968 abdominal surgery patients, they found that 13.53% reported CPSP 1 year post-surgery. A myriad of risk factors—including age, surgery type and duration, postoperative neutrophil count and NLR, and others—correlated with heightened CPSP risk. Critically, a NLR change ratio (post-surgery vs. pre-surgery) of ≥ 5 was closely linked with CPSP, intensity of pain, and diminished quality of life. Additionally, Chen et al. delved into the predictive merit of the monocyte-lymphocyte ratio concerning postoperative acute kidney injury and patient outcomes among those with aortic dissection. Their findings illuminate the potential of the monocyte-lymphocyte ratio as a viable biomarker for postoperative acute kidney injury and overall patient prognosis.

The effects of anaerobic drugs on organ functions has emerged as a pivotal area of investigation in perioperative research. Wei et al. appraised the effectiveness of dexamethasone and diazepam as supplemental agents in epidural anesthesia for arthroscopic shoulder procedures. Their findings indicate that both pharmaceuticals extend the analgesic duration and curtail medication intake, with dexamethasone exhibiting pronounced superiority. Zhang and Yin embarked on an exploration of how anesthetics might influence postoperative cognitive dysfunction. Their research unveiled that anesthetics possess the capacity to elicit both anti-inflammatory and pro-inflammatory responses, chiefly through modulating microglial activity. Additionally, Meng et al. examined the repercussions of administering butorphanol prior to anesthesia on the incidence of emergence agitation in patients subjected to thoracic operations. Their data suggests that butorphanol administration is efficacious in diminishing the onset of emergence agitation among thoracic surgery recipients, thereby enhancing post-surgical outcomes.

In summary, by delving into the study of multi-organ dysfunction during critical illnesses and the post-anesthesia diagnosis, we can better identify and mitigate complications, notably perioperative organ damage. This enhances both the therapeutic outcomes and survival rates of perioperative patients, holding significant value for early intervention and prevention.

## Author contributions

RL: Writing—review & editing. QG: Writing—review & editing. JM: Writing—review & editing. CD: Writing—original draft, Writing—review & editing.

